# Comparison of different membrane supports for monolayer culture of bovine oviduct epithelial cells

**DOI:** 10.1186/1753-6561-5-S1-P117

**Published:** 2011-11-22

**Authors:** Muhammad Z Tahir, Fabien George, Isabelle Donnay

**Affiliations:** 1 UMR-BDR 1198 INRA/ENVA, Ecole Nationale Vétérinaire d’Alfort, 94700 Maisons-Alfort, France; 2 ISV-EMCA, Université Catholique de Louvain, 1348 Louvain-la-Neuve, Belgium

## Background

The oviduct epithelium consists of ciliated and secretory cells which play an important role in key reproductive processes such as sperm capacitation, fertilization and early embryonic development. Bovine oviduct epithelial cells have been widely used in co-culture experiments to condition culture media and improve early embryonic development [1]. However, these cells dedifferentiate during *in vitro* culture and manifest alterations like loss of cilia and secretory granules, reduction of cell height and flattening on the culture surface [2]. The trials for long term culture of oviduct cells, ensuring a better polarization and differentiation of the cells, include culture of oviduct cells on matrigel supports or collagen filter inserts. In this study, we have compared three different membrane supports for their potential to maintain ultrastructural features and monolayer integrity of bovine oviduct epithelial cells during *in vitro* culture.

## Materials and methods

Oviducts were excised from the genital tracts of cows slaughtered in abattoir. Following mechanical isolation, the oviduct epithelial cells were cultured on three different membrane supports i.e. polyester membrane (Thincert™, Millipore Inc. USA), polytetrafluoroethylene membrane (Transwell™, Corning Inc. USA) and cellulose ester membrane (Millicell™, Millipore Inc. USA). The culture of oviduct epithelial cells was done in TCM-199 medium (Gibco, Grand Island, NY, USA) with 10% fetal bovine serum at 39°C in a humidified atmosphere of 5% CO_2_ & 20% O_2_. The development of primary cultures was assessed daily and the medium was renewed every 48 hours. After 3 weeks of monolayer culture, the ultrastructural features of oviduct cells were examined by scanning electron microscopy while the integrity of monolayer was tested by transepithelial electrical resistance and permeability to radiolabeled ^14^C-Mannitol. Non-parametric statistical analysis was done using Kurskal-Wallis Test.

## Results

**Figure 1:**
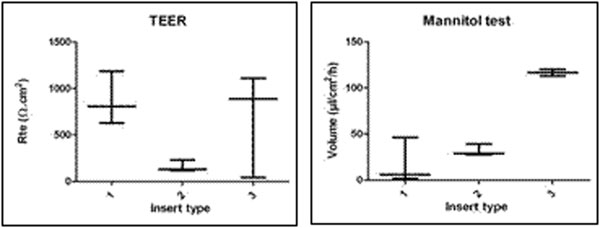
1a) TEER (transepithelial electrical resistance) across oviduct cells monolayer (median ± extreme values) on 1. Thincert™, 2. Transwell™, 3. Millicell™ inserts. 1b) Mannitol test (permeability to radioactive mannitol) through oviduct cells monolayer (median ± extreme values) on 1. Thincert™, 2. Transwell™, 3. Millicell™ inserts.

The study of ultrastructural features by scanning electron microscopy revealed presence of an intact monolayer of polygonal epithelial cells on polyester (Thincert™) and cellulose ester (Millicell™) membrane supports while no such monolayer was seen in polytetrafluoroethylene (Transwell™) membrane support. The test of transepithelial electrical resistance across monolayer of oviduct epithelial cells showed no significant difference (P>0.25) between the three membrane supports (Fig. 1a). The test for permeability of radiolabeled ^14^C-Mannitol across the monolayer of oviduct epithelial cells showed a tendency for lowest permeability (P=0.6) in polyester (Thincert™) membrane support (Fig. 1b).

## Conclusion

The potential of polyester membrane supports (Thincert™) to maintain ultrastructural features (intact monolayer of polygonal epithelial cells) and ensure monolayer integrity (higher transepithelial electrical resistance and lowest permeability to mannitol) during *in vitro* culture may serve as a model to study different cellular functions such as transport, absorption and secretory capacity of bovine oviduct epithelial cells as well as embryo-maternal interactions.
